# SrZnSi_3_O_8_, a synthetic member of the feldspar group

**DOI:** 10.1107/S2056989026005852

**Published:** 2026-06-05

**Authors:** Matthias Weil

**Affiliations:** a Institute for Chemical Technologies and Analytics, Division of Applied Solid State Chemistry, Getreidemarkt 9/E164-05-1, 1060 Vienna, Austria; University of Massachusetts Dartmouth, USA

**Keywords:** crystal structure, structural comparison, framework structure, diadochy, Zn in tetra­hedral coordination

## Abstract

Strontium *tecto*-zincotrisilicate, SrZnSi_3_O_8_, is a synthetic member of the feldspar group with monoclinic symmetry (space group *P*2_1_/*n*, *Z* = 4). In comparison with the alkali feldspars, the aluminium-richest tetra­hedral site in these minerals is completely substituted by Zn, and the alkali metal *M* site by strontium. The crystal structure is fully ordered, *i.e.* there is no mutual co-occupation of the tetra­hedral sites or positional disorder of the *M* site. Although the tetra­hedral site substituted by Zn exhibits significantly longer bond lengths [(Zn—O) = 1.973 Å] due to the incorporation of the larger cation, there is no noticeable distortion of the SiO_4_ tetra­hedra compared to the aluminium-bearing feldspars. The coordination number of Sr^2+^ is [6 + 2], with six short distances to oxygen sites in a narrow range [ = 2.560 Å] and two significantly longer distances > 3.10 Å.

## Abstract 2

Synthetic SrZnSi_3_O_8_ belongs to the feldspar group with 1/4 of the cationic sites of the tetra­hedral framework fully occupied by Zn^2+^ and 3/4 fully occupied by Si^4+^.

## 1. Chemical context

Strontium silicate, Sr_2_SiO_4_, is a well-known host material for luminescence applications. When lanthanides, especially Eu, are used as dopants, efficient white light-emitting diodes can be produced (Park *et al.*, 2003 ▸; Gupta *et al.*, 2015 ▸).

In this context, it was investigated how the luminescence properties change when the Eu-doped host material is modified by incorporating divalent cations (*M* = Ca, Ba, Mg, Zn) to form a possible solid solution (Sr_1.9_*M*_0.1_)SiO_4_ (Wieser, 2006 ▸). In one of these experiments for the intended preparation of (Sr_1.9_Zn_0.1_)SiO_4_:Eu^3+^, SrZnSi_3_O_8_ has been obtained serendipitously instead. Its feldspar-type crystal structure is presented and discussed in the present article.

## 2. Structural commentary

The probable existence of SrZnSi_3_O_8_ was predicted some time ago (Fehr & Huber, 2001 ▸), and this phase was actually obtained during investigations of solid solutions (Ba_1–*x*_Sr_*x*_)ZnSi_3_O_8_ investigated for microwave dielectric properties (Song *et al.*, 2019 ▸). Another phase in the system SrO/Al_2_O_3_/SiO_2_ has been identified to date, *viz*. synthetic Sr-hardysonite, Sr_2_ZnSi_2_O_7_ (Ardit *et al.*, 2010 ▸). The putative crystal structure of SrZnSi_3_O_8_ was derived from laboratory powder X-ray diffraction data and reported as triclinic with space group *P*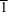 (Song *et al.*, 2019 ▸). Although the corresponding article states: ‘The lattice parameters of [⋯] SrZnSi_3_O_8_ were extracted from XRD data using the least-squares method. All the peaks are indexed accordingly, and the crystal structure information is given in the supplemental file’, no such data are available. Therefore, no direct comparison can be made with the results of the current single crystal X-ray diffraction data. In any case, the latter data clearly revealed that the symmetry is monoclinic rather than triclinic as previously reported. The crystal structure shows a feldspar-type arrangement of the tetra­hedral framework and the metal position.

**Figure 1:**
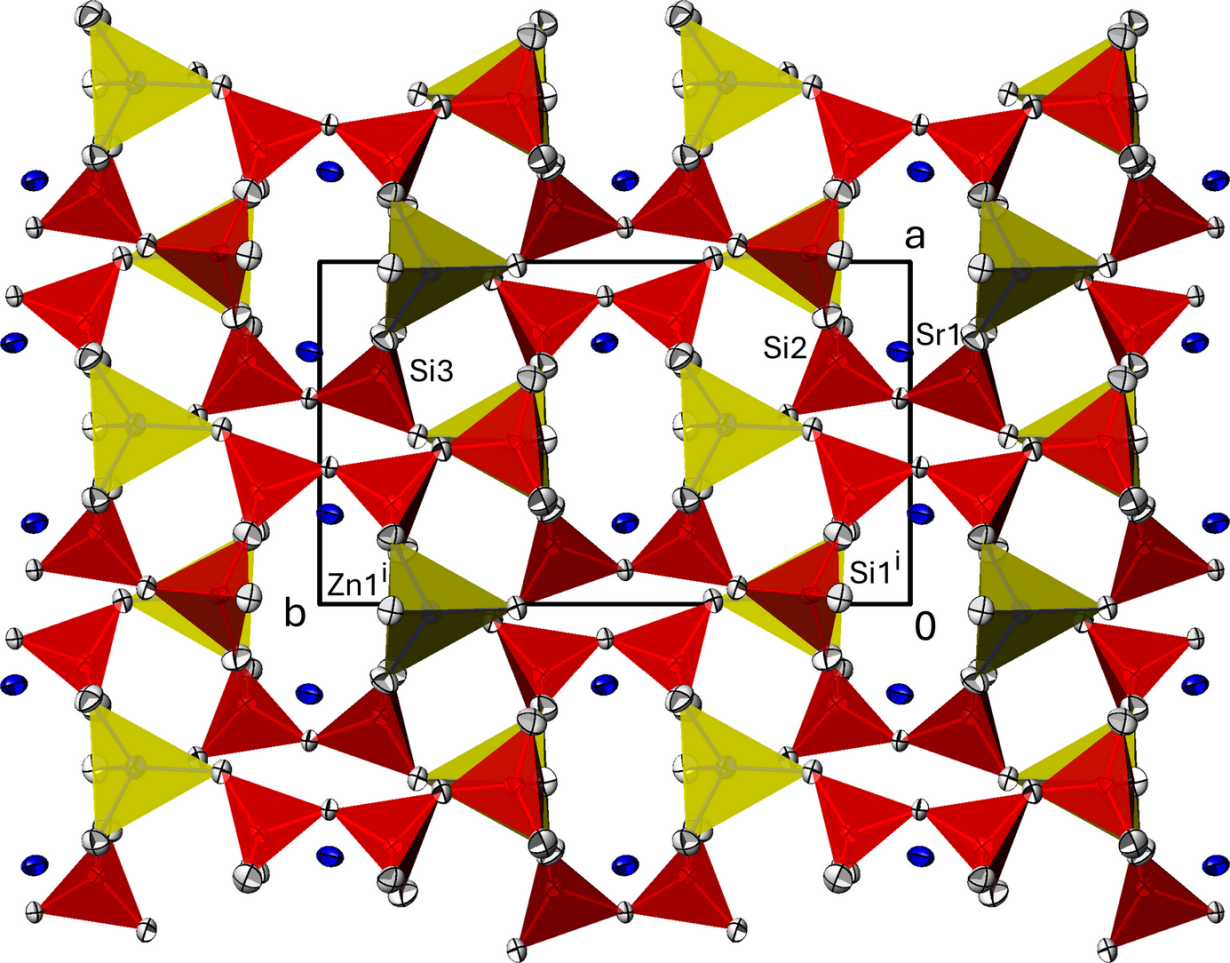
The crystal structure of SrZnSi_3_O_8_ in a projection along [00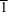]. Displacement ellipsoids are drawn at the 90% probability level; the tetra­hedral framework atoms are shown in polyhedral representation (Zn yellow, Si red). [Symmetry code: (i) −*x*, 1 − *y*, *z*.]

Feldspars define a large group of aluminium silicate minerals (class of tectosilicates; Liebau, 1985 ▸) and are considered the most important rock-forming minerals in the Earth’s crust, accounting for almost 60% of its composition. The frequently occurring alkali (*M*) feldspars can be described with the formula *M*[Al(Al,Si)_3_O_8_], or more generally *M*[*T*_4_O_8_] (where *T* is a tetra­hedrally coordinated site), and crystallize either in the triclinic or monoclinic crystal system with unit cell parameter of *a* ≃ 8.4, *b* ≃ 13.0, *c* ≃ 7.2 Å, *α* ≃ 90, *β* ≃ 116, *c* ≃ 90° (Smith, 1974 ▸). It is precisely this dimension of the unit cell that is also found in the crystal structure of SrZnSi_3_O_8_. This is a case of diadochy, and the tetra­hedral position T_1_(0) (for atomic designations in feldspars, see: Smith, 1974 ▸) is completely occupied by Zn, and Zn also does not co-occupy the other tetra­hedral sites (Fig. 1 ▸). In feldspars, the T_1_(0) position of the tetra­hedral framework is usually occupied by the majority of Al^3+^ present in the structure, i.e. the cation with a lower charge than Si^4+^. In simplified terms, this trend continues in SrZnSi_3_O_8_ with the even lower charged Zn^2+^, and electron neutrality in the overall structure is ensured by the doubly charged alkaline earth metal Sr^2+^, which occupies the *M* site. This substitution at the T_1_(0) site with a considerably larger cation (0.60 Å for Zn^2+^*versus* 0.39 Å for Al^3+^, Shannon, 1976 ▸) results in longer T_1_(0)—O bonds (average 1.973 Å, Table 1 ▸). Compared with the alkaline earth homologues CaZnSi_3_O_8_ (Heuer *et al.*, 1998 ▸) and BaZnSi_3_O_8_ (Zou *et al.*, 2021 ▸), which also exhibit feldspar-like crystal structures, the Zn—O distances are similar. CaZnSi_3_O_8_: 1.916 (3), 1.941 (3), 1.943 (3), 2.047 (3) Å [*P*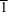, *a* = 8.121 (1), *b* = 12.927 (1), *c* = 7.206 (1) Å, *α* = 93.76 (5), *β* = 116.120 (7), *γ* = 84.368 (7)°, *Z* = 2, single crystal X-ray data, distances calculated from the deposited crystallographic information file (CIF), entry 409286 in the Inorganic Crystal Structure Database (ICSD; Zagorac *et al.*, 2019 ▸)]; BaZnSi_3_O_8_: 1.875 (12), 1.923 (9), 1.972 (11), 1.980 (10) Å [*P*2_1_/*a*, *a* = 8.725 (10), *b* = 13.072 (20), *c* = 7.307 (10) Å, *β* = 115.85 (2)°, *Z* = 4, powder synchrotron X-ray data, distances taken from the publication: note that in this publication and the corresponding deposited CIF, entry 113904 in the ICSD, the fractional coordinates of the Zn site are incorrect, with *x* = 0.012 most likely the correct value].

The three SiO_4_ tetra­hedra in the remaining framework of SrZnSi_3_O_8_ are not significantly distorted and show the usual Si—O bond lengths distributions (Table 1 ▸; averaged values: Si1 1.609, Si2 1.616, Si3 1.621 Å), in very good agreement with the mean bond length of 1.62 Å for an SiO_4_ tetra­hedron (Liebau, 1985 ▸).

**Figure 2:**
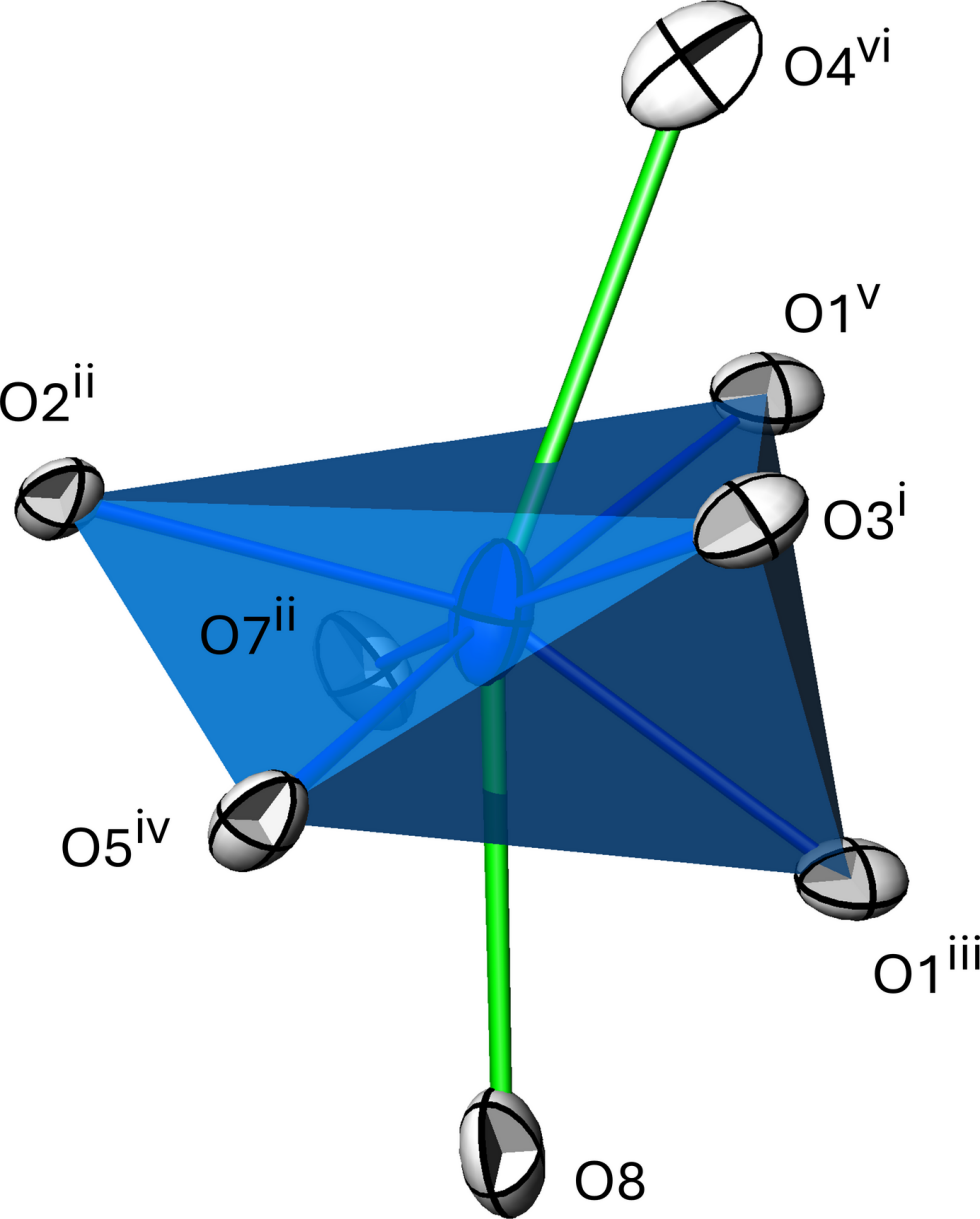
Coordination environment of the Sr1 site. Displacement ellipsoids are drawn at the 90% probability level; symmetry codes refer to Table 1 ▸. The polyhedron includes the six short Sr—O bonds, and the two O atoms capping the polyhedron are shown with green bonds.

The coordination number (CN) of Sr can be described as [6 + 2], with six closer distances in a narrow range (average 2.560 Å) and two considerably longer Sr—O distances > 3.10 Å (Table 1 ▸). The closest matching polyhedron for CN = 6 was calculated with the *Polynator* program (Link & Niewa, 2023 ▸) and is a twisted trigonal prism (idealized point group 32, deviation from idealized values *δ* = 13.690), and the two O atoms at longer distances cap opposite faces (Fig. 2 ▸).

The plausibility of the SrZnSi_3_O_8_ structure model was verified and confirmed using bond-valence-sum calculations (Brown, 2002 ▸) performed with the *ECoN21* program (Ilinca, 2022 ▸). The calculated bond-valence sums (Table 2 ▸) are close to the expected values, and the global instability index (GII) of 0.10 valence units indicates a stable and not particularly strained crystal structure (Salinas-Sanchez *et al.*, 1992 ▸; Brown, 2009 ▸).

**Figure 3:**
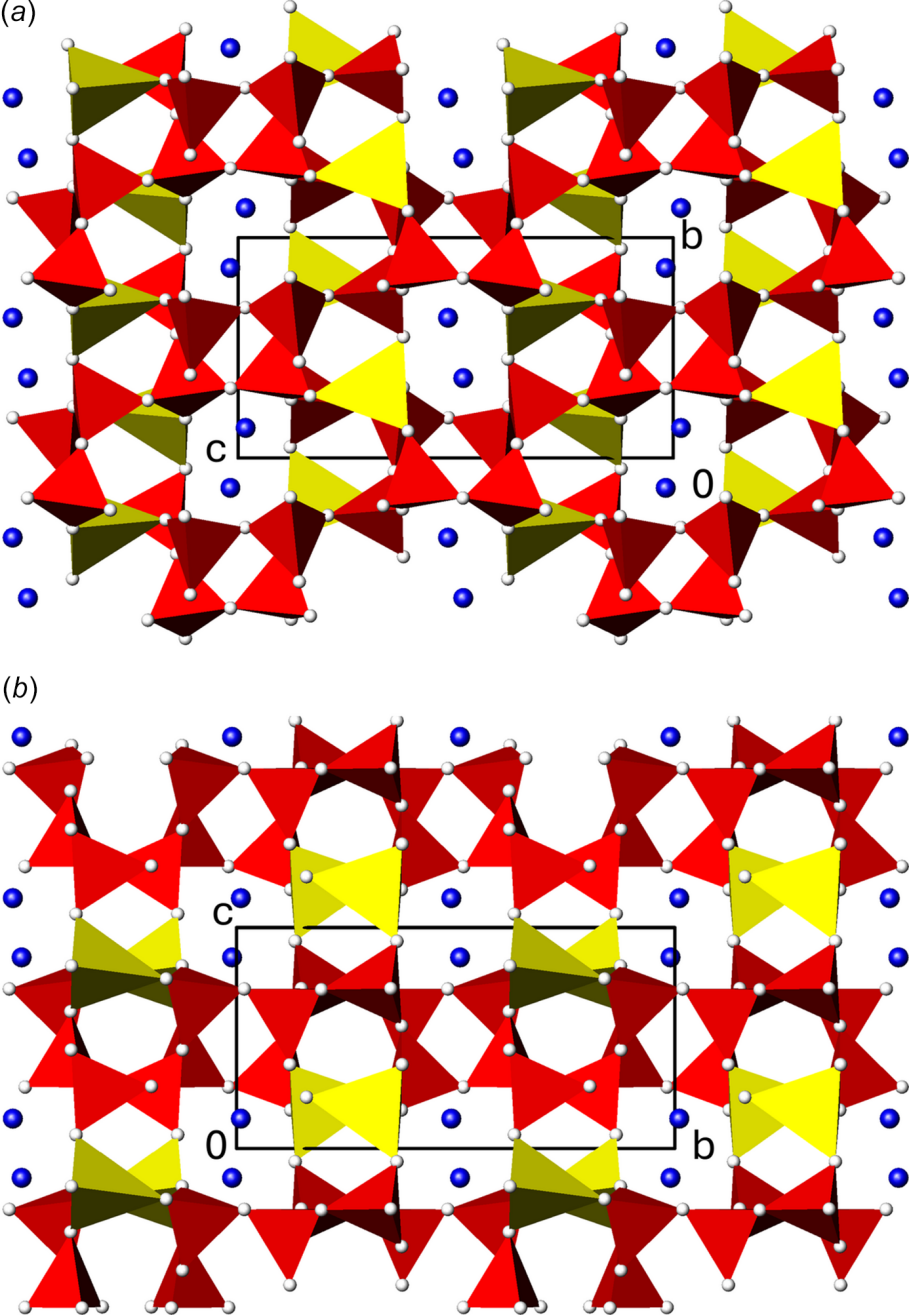
Comparison of the crystal structures of SrZnSi_3_O_8_ (*a*), projection along [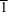
00]) and BaZnSi_3_O_8_ (*b*), projection along [100]). Alkaline earth cations are shown as blue spheres, ZnO_4_ tetra­hedra are yellow and SiO_4_ tetra­hedra are red.

The same formula type, the same space group type and similar lattice parameters might suggest that SrZnSi_3_O_8_ and BaZnSi_3_O_8_ are isotypic to one another. However, a closer look at the crystal structures (under consideration of the corrected *x* parameter for Zn1 in the BaZnSi_3_O_8_ structure, see above) reveals that the two crystalline phases are isopointal (Lima-de-Faria *et al.*, 1990 ▸). As shown in Fig. 3 ▸, the crystal structures are clearly distinct from one another, with a different arrangement of the alkaline earth metal sites and the positions of ZnO_4_ tetra­hedra within the tetra­hedral framework.

## 3. Synthesis and crystallization

SiO_2_ (Fluka, purum), SrCO_3_ (Merck, pure) and ZnO were weighted according to a composition of Sr_1.9_Zn_0.1_SiO_4_ (total 2 g). During intensive mixing of the powders in an achate mortar, small amounts of EuF_3_ (Aldrich, 99+; approx. 10 mg) were added as a dopant. The mixture was then compressed to a pellet that was heated in a corundum crucible at 1543 K for one day. After the reaction, the slightly yellowish sample appeared glassy in some places. A small colourless single crystal of the title compound was extracted from this matrix.

## 4. Refinement

Crystal data, data collection and structure refinement details are summarized in Table 3 ▸. The non-reduced setting of the unit cell was used to emphasize the relationship to the crystal structures of other feldspar group minerals (e.g. microcline, low-albite, high-albite, reedmergernite) listed by Smith (1974 ▸). Similarly, the atomic labelling [Zn1 = T_1_(0), Si1 = T_1_(m), Si2 = T_2_(0), Si3 = T_2_(m), O1 = O_A_(1), O2 = O_A_(2), O3 = O_B_(0), O4 = O_B_(m), O5 = O_C_(0), O6 = O_C_(m), O7 = O_D_(0), O8 = O_D_(m)] and atomic coordinates were also adjusted to the atomic parameters listed there. In order to check for any possible co-occupation of Zn and Si (and *vice versa*), the site occupation factor (s.o.f.) of each Zn1, Si1, Si2 and Si3 site was freely refined (constraining all other sites to be fully occupied). In all cases, the free refinement converged at s.o.f. values very close to 1.0, showing no co-occupation.

## Supplementary Material

Crystal structure: contains datablock(s) I. DOI: 10.1107/S2056989026005852/yy2023sup1.cif

Structure factors: contains datablock(s) I. DOI: 10.1107/S2056989026005852/yy2023Isup2.hkl

CCDC reference: 2559165

Additional supporting information:  crystallographic information; 3D view; checkCIF report

## Figures and Tables

**Table 1:** Selected bond lengths (Å)

Sr1—O3^i^	2.510 (2)	Si1—O1^iii^	1.597 (2)
Sr1—O7^ii^	2.523 (2)	Si1—O4^vii^	1.610 (2)
Sr1—O1^iii^	2.533 (2)	Si1—O6	1.613 (2)
Sr1—O2^ii^	2.583 (2)	Si1—O8	1.614 (2)
Sr1—O5^iv^	2.593 (2)	Si2—O3	1.580 (2)
Sr1—O1^v^	2.617 (2)	Si2—O6^ix^	1.621 (2)
Sr1—O8	3.115 (3)	Si2—O8^x^	1.624 (2)
Sr1—O4^vi^	3.294 (3)	Si2—O2	1.638 (2)
Zn1—O5	1.903 (2)	Si3—O7^x^	1.587 (2)
Zn1—O7	1.920 (2)	Si3—O5^iv^	1.601 (2)
Zn1—O3^vii^	1.969 (2)	Si3—O4	1.630 (3)
Zn1—O1^viii^	2.010 (2)	Si3—O2^ii^	1.665 (2)

Symmetry codes: (i) 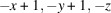; (ii) 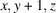; (iii) 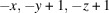; (iv) 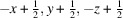; (v) 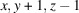; (vi) 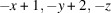; (vii) 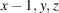; (viii) 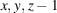; (ix) 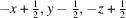; (x) 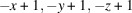.

**Table 2:** Bond-valence-sum calculations (in valence units) for SrZnSi_3_O_8_

	Sr1	Zn1	Si1	Si2	Si3	Σ
O1	0.30, 0.25	0.43	1.07			2.05
O2	0.27			0.96	0.90	2.13
O3	0.32	0.47		1.12		1.91
O4	0.06		1.04		0.99	2.09
O5	0.27	0.56			1.06	1.89
O6			1.03	1.01		2.04
O7	0.31	0.54			1.10	1.95
O8	0.09		1.03	1.00		2.12
Σ	1.87	2.00	4.17	4.09	4.05	

**Table 3:** Experimental details

Crystal data	
Chemical formula	SrZnSi_3_O_8_
*M*_r_	365.26
Crystal system, space group	Monoclinic, *P*2_1_/*n*
Temperature (K)	293
*a*, *b*, *c* (Å)	8.3060 (8), 13.0111 (13), 7.2454 (7)
β (°)	114.822 (2)
*V* (Å^3^)	710.67 (12)
*Z*	4
Radiation type	Mo *K*α
μ (mm^−1^)	11.40
Crystal size (mm)	0.06 × 0.04 × 0.03

Data collection	
Diffractometer	Bruker SMART APEXII CCD
Absorption correction	Multi-scan (*SADABS*; Krause *et al.*, 2015 ▸)
*T*_min_, *T*_max_	0.548, 0.764
No. of measured, independent and observed [*I* > 2σ(*I*)] reflections	7658, 2057, 1745
*R*_int_	0.035
(sin θ/λ)_max_ (Å^−1^)	0.703

Refinement	
*R*[*F*^2^ > 2σ(*F*^2^)], *wR*(*F*^2^), *S*	0.029, 0.069, 1.03
No. of reflections	2057
No. of parameters	118
Δρ_max_, Δρ_min_ (e Å^−3^)	0.88, −0.67

Computer programs: *APEX2* and *SAINT* (Bruker, 2005 ▸), *SHELXS* (Sheldrick, 2008 ▸), *SHELXL* (Sheldrick, 2015 ▸), *ATOMS* (Dowty, 2006 ▸) and *publCIF* (Westrip, 2010 ▸).

## supplementary crystallographic information

### Strontium tecto-zincotrisilicate Crystal data

**Table:**

SrZnSi_3_O_8_	*F*(000) = 696
*M**_r_* = 365.26	*D*_x_ = 3.414 Mg m^−^^3^
Monoclinic, *P*2_1_/*n*	Mo *K*α radiation, λ = 0.71073 Å
*a* = 8.3060 (8) Å	Cell parameters from 2419 reflections
*b* = 13.0111 (13) Å	θ = 3.1–30.0°
*c* = 7.2454 (7) Å	µ = 11.40 mm^−^^1^
β = 114.822 (2)°	*T* = 293 K
*V* = 710.67 (12) Å^3^	Fragment, colourless
*Z* = 4	0.06 × 0.04 × 0.03 mm

### Strontium tecto-zincotrisilicate Data collection

**Table:**

Bruker SMART APEXII CCD diffractometer	2057 independent reflections
Radiation source: fine-focus sealed tube	1745 reflections with *I* > 2σ(*I*)
Graphite monochromator	*R*_int_ = 0.035
ω–scans	θ_max_ = 30.0°, θ_min_ = 3.1°
Absorption correction: multi-scan (SADABS; Krause *et al.*, 2015)	*h* = −11→11
*T*_min_ = 0.548, *T*_max_ = 0.764	*k* = −18→18
7658 measured reflections	*l* = −10→9

### Strontium tecto-zincotrisilicate Refinement

**Table:**

Refinement on *F*^2^	118 parameters
Least-squares matrix: full	0 restraints
*R*[*F*^2^ > 2σ(*F*^2^)] = 0.029	*w* = 1/[σ^2^(*F*_o_^2^) + (0.0377*P*)^2^ + 0.2907*P*] where *P* = (*F*_o_^2^ + 2*F*_c_^2^)/3
*wR*(*F*^2^) = 0.069	(Δ/σ)_max_ = 0.001
*S* = 1.03	Δρ_max_ = 0.88 e Å^−^^3^
2057 reflections	Δρ_min_ = −0.67 e Å^−^^3^

### Strontium tecto-zincotrisilicate Special details

**Table:**

Geometry. All esds (except the esd in the dihedral angle between two l.s. planes) are estimated using the full covariance matrix. The cell esds are taken into account individually in the estimation of esds in distances, angles and torsion angles; correlations between esds in cell parameters are only used when they are defined by crystal symmetry. An approximate (isotropic) treatment of cell esds is used for estimating esds involving l.s. planes.

### Strontium tecto-zincotrisilicate Fractional atomic coordinates and isotropic or equivalent isotropic displacement parameters (Å^2^)

**Table:**

	*x*	*y*	*z*	*U*_iso_*/*U*_eq_	
Sr1	0.26316 (4)	0.98248 (2)	0.13696 (5)	0.01620 (10)	
Zn1	0.02329 (5)	0.18835 (3)	0.20052 (6)	0.01043 (10)	
Si1	−0.02817 (11)	0.83033 (6)	0.24716 (13)	0.00802 (17)	
Si2	0.70774 (11)	0.12373 (7)	0.32678 (13)	0.00844 (17)	
Si3	0.67150 (11)	0.89689 (7)	0.38177 (13)	0.00864 (17)	
O1	0.0187 (3)	0.11994 (18)	0.9496 (3)	0.0128 (5)	
O2	0.6002 (3)	0.01681 (16)	0.3169 (3)	0.0103 (4)	
O3	0.8042 (3)	0.12069 (18)	0.1797 (4)	0.0147 (5)	
O4	0.8007 (3)	0.87771 (19)	0.2669 (4)	0.0191 (5)	
O5	0.0104 (3)	0.33381 (17)	0.2183 (4)	0.0132 (5)	
O6	−0.0480 (3)	0.70691 (17)	0.2380 (4)	0.0137 (5)	
O7	0.2234 (3)	0.10919 (19)	0.3782 (3)	0.0163 (5)	
O8	0.1561 (3)	0.85914 (19)	0.4367 (4)	0.0175 (5)	

### Strontium tecto-zincotrisilicate Atomic displacement parameters (Å^2^)

**Table:**

	*U*^11^	*U*^22^	*U*^33^	*U*^12^	*U*^13^	*U*^23^
Sr1	0.00884 (14)	0.01500 (16)	0.02113 (18)	0.00124 (11)	0.00275 (12)	−0.00635 (12)
Zn1	0.01131 (18)	0.00930 (18)	0.01112 (19)	0.00110 (13)	0.00514 (14)	−0.00009 (13)
Si1	0.0077 (4)	0.0086 (4)	0.0086 (4)	−0.0006 (3)	0.0042 (3)	−0.0005 (3)
Si2	0.0078 (4)	0.0080 (4)	0.0093 (4)	−0.0005 (3)	0.0034 (3)	0.0001 (3)
Si3	0.0076 (4)	0.0074 (4)	0.0112 (4)	0.0010 (3)	0.0042 (3)	0.0002 (3)
O1	0.0173 (11)	0.0135 (11)	0.0105 (11)	−0.0011 (9)	0.0088 (9)	0.0003 (8)
O2	0.0093 (10)	0.0062 (10)	0.0142 (11)	−0.0005 (8)	0.0039 (9)	0.0019 (8)
O3	0.0148 (11)	0.0156 (11)	0.0177 (12)	−0.0021 (9)	0.0107 (10)	−0.0003 (9)
O4	0.0181 (12)	0.0172 (12)	0.0294 (14)	0.0011 (10)	0.0172 (11)	−0.0038 (10)
O5	0.0118 (10)	0.0082 (10)	0.0191 (12)	0.0012 (8)	0.0061 (9)	0.0002 (9)
O6	0.0123 (11)	0.0083 (10)	0.0218 (12)	−0.0014 (8)	0.0085 (10)	−0.0011 (9)
O7	0.0157 (11)	0.0167 (12)	0.0124 (12)	0.0046 (9)	0.0019 (10)	0.0025 (9)
O8	0.0155 (11)	0.0176 (12)	0.0128 (12)	−0.0026 (9)	−0.0006 (10)	−0.0031 (9)

### Strontium tecto-zincotrisilicate Geometric parameters (Å, º)

**Table:**

Sr1—O3^i^	2.510 (2)	Si1—O1^iii^	1.597 (2)
Sr1—O7^ii^	2.523 (2)	Si1—O4^vii^	1.610 (2)
Sr1—O1^iii^	2.533 (2)	Si1—O6	1.613 (2)
Sr1—O2^ii^	2.583 (2)	Si1—O8	1.614 (2)
Sr1—O5^iv^	2.593 (2)	Si2—O3	1.580 (2)
Sr1—O1^v^	2.617 (2)	Si2—O6^ix^	1.621 (2)
Sr1—O8	3.115 (3)	Si2—O8^x^	1.624 (2)
Sr1—O4^vi^	3.294 (3)	Si2—O2	1.638 (2)
Zn1—O5	1.903 (2)	Si3—O7^x^	1.587 (2)
Zn1—O7	1.920 (2)	Si3—O5^iv^	1.601 (2)
Zn1—O3^vii^	1.969 (2)	Si3—O4	1.630 (3)
Zn1—O1^viii^	2.010 (2)	Si3—O2^ii^	1.665 (2)
			
O3^i^—Sr1—O7^ii^	159.85 (8)	O6—Si1—O8	108.09 (13)
O3^i^—Sr1—O1^iii^	70.38 (7)	O3—Si2—O6^ix^	113.99 (13)
O7^ii^—Sr1—O1^iii^	98.00 (8)	O3—Si2—O8^x^	112.93 (13)
O3^i^—Sr1—O2^ii^	109.40 (7)	O6^ix^—Si2—O8^x^	109.65 (14)
O7^ii^—Sr1—O2^ii^	88.09 (7)	O3—Si2—O2	111.75 (13)
O1^iii^—Sr1—O2^ii^	155.38 (7)	O6^ix^—Si2—O2	100.91 (12)
O3^i^—Sr1—O5^iv^	79.41 (8)	O8^x^—Si2—O2	106.74 (13)
O7^ii^—Sr1—O5^iv^	119.40 (7)	O7^x^—Si3—O5^iv^	116.82 (14)
O1^iii^—Sr1—O5^iv^	98.38 (7)	O7^x^—Si3—O4	112.02 (14)
O2^ii^—Sr1—O5^iv^	58.43 (7)	O5^iv^—Si3—O4	112.87 (13)
O3^i^—Sr1—O1^v^	93.71 (7)	O7^x^—Si3—O2^ii^	108.85 (13)
O7^ii^—Sr1—O1^v^	67.31 (7)	O5^iv^—Si3—O2^ii^	101.33 (12)
O1^iii^—Sr1—O1^v^	78.22 (8)	O4—Si3—O2^ii^	103.38 (13)
O2^ii^—Sr1—O1^v^	125.74 (7)	Si1^iii^—O1—Zn1^xi^	129.67 (14)
O5^iv^—Sr1—O1^v^	173.05 (7)	Si1^iii^—O1—Sr1^iii^	112.26 (11)
O3^i^—Sr1—O8	110.02 (7)	Zn1^xi^—O1—Sr1^iii^	96.98 (9)
O7^ii^—Sr1—O8	72.24 (7)	Si1^iii^—O1—Sr1^xii^	114.94 (12)
O1^iii^—Sr1—O8	52.92 (6)	Zn1^xi^—O1—Sr1^xii^	96.74 (8)
O2^ii^—Sr1—O8	107.66 (7)	Sr1^iii^—O1—Sr1^xii^	101.78 (8)
O5^iv^—Sr1—O8	72.81 (7)	Si2—O2—Si3^xiii^	131.35 (14)
O1^v^—Sr1—O8	109.11 (7)	Si2—O2—Sr1^xiii^	128.63 (11)
O3^i^—Sr1—O4^vi^	65.95 (7)	Si3^xiii^—O2—Sr1^xiii^	99.33 (10)
O7^ii^—Sr1—O4^vi^	103.62 (7)	Si2—O3—Zn1^xiv^	130.61 (14)
O1^iii^—Sr1—O4^vi^	106.45 (7)	Si2—O3—Sr1^i^	130.42 (13)
O2^ii^—Sr1—O4^vi^	95.10 (7)	Zn1^xiv^—O3—Sr1^i^	98.83 (9)
O5^iv^—Sr1—O4^vi^	126.17 (7)	Si1^xiv^—O4—Si3	154.04 (19)
O1^v^—Sr1—O4^vi^	50.31 (6)	Si1^xiv^—O4—Sr1^vi^	87.21 (11)
O8—Sr1—O4^vi^	156.53 (6)	Si3—O4—Sr1^vi^	118.31 (12)
O5—Zn1—O7	123.07 (10)	Si3^ix^—O5—Zn1	123.27 (13)
O5—Zn1—O3^vii^	111.92 (10)	Si3^ix^—O5—Sr1^ix^	100.79 (10)
O7—Zn1—O3^vii^	108.89 (10)	Zn1—O5—Sr1^ix^	135.84 (11)
O5—Zn1—O1^viii^	121.42 (10)	Si1—O6—Si2^iv^	137.20 (16)
O7—Zn1—O1^viii^	92.94 (10)	Si3^x^—O7—Zn1	133.22 (15)
O3^vii^—Zn1—O1^viii^	93.82 (10)	Si3^x^—O7—Sr1^xiii^	123.71 (13)
O1^iii^—Si1—O4^vii^	107.47 (13)	Zn1—O7—Sr1^xiii^	102.37 (10)
O1^iii^—Si1—O6	114.14 (13)	Si1—O8—Si2^x^	157.11 (19)
O4^vii^—Si1—O6	107.82 (13)	Si1—O8—Sr1	88.43 (10)
O1^iii^—Si1—O8	106.32 (13)	Si2^x^—O8—Sr1	112.89 (12)
O4^vii^—Si1—O8	113.13 (14)		

Symmetry codes: (i) −*x*+1, −*y*+1, −*z*; (ii) *x*, *y*+1, *z*; (iii) −*x*, −*y*+1, −*z*+1; (iv) −*x*+1/2, *y*+1/2, −*z*+1/2; (v) *x*, *y*+1, *z*−1; (vi) −*x*+1, −*y*+2, −*z*; (vii) *x*−1, *y*, *z*; (viii) *x*, *y*, *z*−1; (ix) −*x*+1/2, *y*−1/2, −*z*+1/2; (x) −*x*+1, −*y*+1, −*z*+1; (xi) *x*, *y*, *z*+1; (xii) *x*, *y*−1, *z*+1; (xiii) *x*, *y*−1, *z*; (xiv) *x*+1, *y*, *z*.
